# The threshold model revisited

**DOI:** 10.1111/jep.13091

**Published:** 2018-12-21

**Authors:** Benjamin Djulbegovic, Iztok Hozo, Thomas Mayrhofer, Jef van den Ende, Gordon Guyatt

**Affiliations:** ^1^ Department of Supportive Care Medicine, Department of Hematology City of Hope National Medical Center Duarte California USA; ^2^ Program for Evidence‐based Medicine and Comparative Effectiveness Research Duarte California USA; ^3^ Department of Mathematics and Actuarial Science Indiana University Northwest Gary Indiana USA; ^4^ Cardiac MR PET CT Program Massachusetts General Hospital & Harvard Medical School Boston Massachusetts USA; ^5^ Faculty of Medicine and Health Sciences University of Antwerp Antwerp Belgium; ^6^ Department of Health Research Methods, Evidence and Impact McMaster University Hamilton Canada

**Keywords:** decision analysis, decision‐making, evidence‐based medicine, threshold model

## Abstract

**Background:**

The threshold model represents one of the most significant advances in the field of medical decision‐making, yet it often does not apply to the most common class of clinical problems, which include health outcomes as a part of definition of disease. In addition, the original threshold model did not take a decision‐maker's values and preferences explicitly into account.

**Methods:**

We reformulated the threshold model by (1) applying it to those clinical scenarios, which define disease according to outcomes that treatment is designed to affect, (2) taking into account a decision‐maker's values.

**Results:**

We showed that when outcomes (eg, morbidity) are integral part of definition of disease, the classic threshold model does not apply (as this leads to double counting of outcomes in the probabilities and utilities branches of the model). To avoid double counting, the model can be appropriately analysed by assuming diagnosis is certain (*P* = 1). This results in deriving a different threshold—the threshold for outcome of disease (*M*_*t*_ *)* instead of threshold for probability of disease (*P*_*t*_ *)* above which benefits of treatment outweigh its harms.

We found that *M*_*t*_ ≤ *P*_*t*_, which may explain differences between normative models and actual behaviour in practice. When a decision‐maker values outcomes related to benefit and harms differently, the new threshold model generates decision thresholds that could be descriptively more accurate.

**Conclusions:**

Calculation of the threshold depends on careful disease versus utility definitions and a decision‐maker's values and preferences.

## 1 INTRODUCTION

The development of threshold model^1^ is considered as one of the most important advances in medical decision‐making.^2^ It is a normative decision model, originally applied to diagnosis that calculates the probability of disease/diagnosis at which a decision‐maker should or ought to choose a treatment when no further diagnostic information is available.^1^ However, the model has not been widely used in clinical practice because, as originally described from the expected utility theory (EUT) point of view, the probability of disease at which we should opt for treatment has typically been considered implausibly different from the actual or descriptive decision‐making thresholds seen in practice.^2^

One possible reason for the difference between behaviour and the threshold model guidance is that people often violate EUT that served as a theoretical framework for derivation of the original threshold model.^3^, ^4^ To address this discrepancy between normative and observed behaviour, subsequent threshold models have been formulated from non‐EUT perspective such as regret, dual processing, and hybrid theoretical stances.^2^

Additional explanations for differences between normative and descriptive estimates generated by the threshold models may, however, exist: (1) descriptive inaccuracy may be a consequence of misapplication of the original threshold model—originally described when diagnosis is not certain when the patient was first seen—to clinical situations when treatment is offered to patients with prior confirmed disease to prevent subsequent events, or disease is defined by the very outcomes treatment is designed to prevent; (2) the original threshold model and subsequent reformulation of the threshold model may not have taken decision‐makers' values and preferences (V&P) explicitly into account.^5^, ^6^, ^7^, ^8^

In this paper, we revisit the EUT threshold model by taking into account these new considerations to modify calculation of the treatment action thresholds. We focus on clinical situation when no further diagnostic tests are available to a decision‐maker to decrease diagnostic uncertainty,^1^ although, as argued below, similar considerations can be applied to clinical settings when such a diagnostic test is available.^9^ We also discuss some common pitfalls in the interpretation of the threshold model aiming to provide needed clarifications for the wider‐clinical applications of this simple but powerful model. In what follows, the terms such as disease events, outcomes, (dis)‐utilities, and risks of morbidity are often used interchangeably (see Appendix for definition).

## 2 METHODS

### 2.1 A brief overview of the threshold model

**Figure 1:**
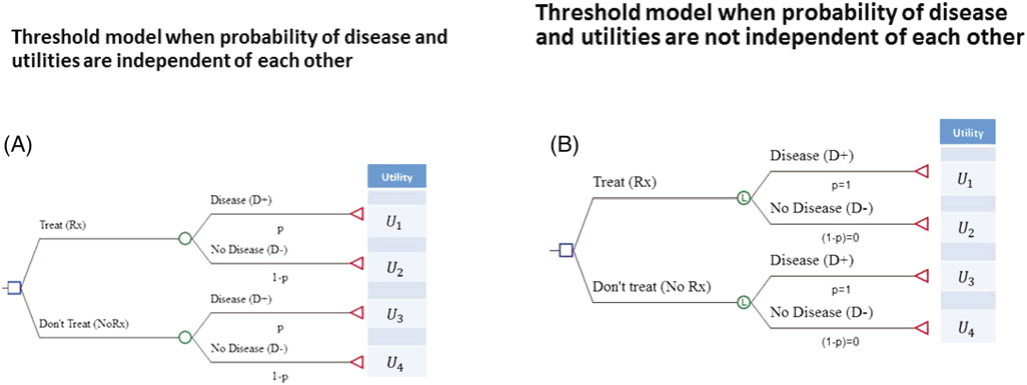
This shows the threshold model for the patients with probability of diagnosis/disease denoted as *P*. A, Probabilities and utilities are independent of each other; B, probabilities and utilities are not independent of each other. Abbreviations: *U*_1_ = *U*[*Rx*, *D*+] utility of health state when treatment is given, and the patient has the disease. Similarly, *U*_2_ = *U*[*Rx*, *D*−] utility of health state when treatment is given and the patient has no disease, *U*_3_ = *U*[*NoRx*, *D*+] utility of health state when no treatment is given and the patient has disease,*U*_4_ = *U*[*NoRx*, *D*−] utility of health state when treatment is not given and the patient has no disease; *P*= probability of disease. See Appendix and text for details

The threshold model (Figure 1) was originally developed to provide an answer to the question: “At which probability of disease (*P*) are we indifferent between expected utility (EU) of administering treatment versus not?^1^”: if *P* is above the threshold probability (*P*_*t*_), a decision‐maker (physician or patient) should choose treatment (Rx), otherwise not (NoRx).

In the classic Pauker & Kassirer (P&K) model^1^ (Figure 1), the expected utilities of each decision are given as follows:

$$\mathrm{EU}\left[\mathrm{Rx}\right]=p\cdot U_{1}+\left(1-p\right)U_{2}\;\mathrm{EU}\left[\text{NoRx}\right]=p\cdot U_{3}+\left(1-p\right)U_{4},$$

Pauker and Kassirer^1^ defined the net benefit of treatment as *B* = *U*_1_ − *U*_3_, ie, the difference in the utility of the outcomes if the diseased patient were treated or were not treated and net harms as *H* = *U*_4_ − *U*_2_, ie, the difference in the utility of the outcomes if a patient without the disease were not treated versus treated. Solving the tree, the threshold probability (*P*_*t*_) above which we should treat is calculated as follows:

(1) $$P_{t}=\frac{1}{1+\frac{B}{H}.}$$

Equation 1 is based on generic definition of net benefits and harms; specific version of the threshold equation depends on the units for utilities (outcomes) used to derive net benefits and net harms^6^ as further outlined below and in Appendix.

### 2.2 Assessing if the probabilities and outcomes (utilities) in the threshold model are independent of each other

Figure 1A shows that key structural ingredients of the threshold decision model compose of the probability of disease (*P*) and consequences of our decisions expressed via health outcomes or our values that we assign to these outcomes (utilities).^1^, ^9^ Each branch in the model is characterized by its probability and outcome (utility, U) with which it is associated.^1^, ^9^ Importantly, probabilities and utilities are assumed to be independent of each other^10^; that is, the value of the utility U remains the same regardless of which value *P* takes. For example, if diagnosis of cancer relies on pathological findings and utilities are based on morbidities or mortality, we can use Equation 1 to calculate the probability of diagnosis of cancer at which treatment benefits outweigh its harms.

However, consider a patient with an actual or presumed condition—or diagnosis—(eg, prior deep venous thrombosis [DVT] or underlying—though possibly as yet undetected—coronary artery disease) that puts the patient at risk of a subsequent morbid or mortal event. The clinician is considering offering an intervention that will decrease the probability of this event occurring. One way of conceptualizing such a situation is that patients do not have the condition of interest until they have the event (eg, they have had a prior DVT, but they only have a new diagnosis once they have a recurrence, and otherwise, they remain “disease free”; patients with presumed or proven underlying coronary artery disease only had a diagnosis when they suffer their subsequent myocardial infarction and otherwise remain “disease free”). Note, these are also clinical situations relating to the secondary prophylaxis, ie, physicians face decision whether to offer treatment to prevent recurrent event, which then defines the very disease that is being treated.

This conceptualization becomes particularly compelling when a particular test result defines the diagnosis and a negative test rules out the diagnosis. This is true for the entire field of thrombosis in which the risk assessment of diagnosis of venous thromboembolism (VTE) is based on detection of what one might conceptualize as “outcomes.” For instance, the widely popular Wells' predictive model for diagnosis of pulmonary embolism (PE) relies on clinical signs and symptoms to establish the pretest probability of PE, which, in turn, is defined by test result of imaging studies (ie, the diagnosis of PE on the test could be conceptualized as the “outcome”).^11^ In cardiovascular field, myocardial infarction is typically defined by a composite outcome that includes a number of tests. Similarly, in oncology, clinicians use imaging or other tests to detect relapse of cancer, metastasis, etc, which is often integral part of definition of disease‐free outcomes. Note, in the case of PE and myocardial infarction, treatment can be conceptualized as primary prophylaxis, ie, it was designed to prevent the disease events before they happened; in the case of prevention of cancer relapse, treatment represents secondary prophylaxis. In either case, disease is defined by events or outcomes that treatment is designed to prevent.

This creates a problem of double counting and violates independence requirement^12^: Morbidity outcomes or events are used both to define probabilities of disease and utilities in the decision tree (Figure 1). For example, if utility is defined as *U* = 1 – *M* (where M = morbidity) (see Appendix), then morbidity changes if probability of disease changes. So, *P* and *U* are no longer independent. This requires asking a different question and calculation of a different threshold than the one based on the classic model (where we assume that *P* and *U* remain independent).

One can express net benefits and harms in various units: as health outcomes related to morbidity or mortality, survival, life expectancy, quality‐adjusted life expectancy, costs, etc. In this paper, we will express net benefits and harms through disutilities related to morbidity or mortality associated with treating versus not treating patients who may have or may have not the disease of interest (in the prior conceptualization with thrombosis and coronary examples, the disease of interest is present only if the patient has a subsequent morbid event) (see also Appendix).

In this article, we refer back to the classical threshold model only to draw comparison to the application of another threshold model that is applicable to the clinical situations when one can conceptualize the disease or condition of interest as being defined by outcomes that treatments are designed to prevent.

### 2.3 Two thresholds

The preceding discussion indicates that the threshold model can generate two thresholds answering two questions: (1) when the probability of disease and probability of outcomes are independent of each other (classic threshold model) and (2) when the probability of disease and probability of outcomes are not independent of each other (an alternative model presented in this paper).

The classic threshold equation^1^, ^9^ (Equation 1) provides the answer to “At which probability of diagnosis/disease should we administer a treatment with given benefit and harms?” This equation should be used when the probability of disease and probability of outcomes are independent of each other (Figure 1A). The model Figure 1A (Equation 1) provides the answer without taking test results into consideration.

When the risk of disease and the risk of outcomes are not independent, one way to avoid double counting is to simply set probability of disease to 1. Thus, from modelling perspective, we assume that disease is certain. In the previous examples, prior DVT establishes a predisposition to recurrence, and this predisposition constitutes the “disease.” After a certain age, we all have some degree of coronary artery disease, and are, thus, at risk of coronary events. This also agrees with clinical and biological logic as the patient cannot develop disease outcome unless he/she has diagnosis or a condition, which causes given outcomes. At the same time, we can never be sure which of the individual patients will have disease outcome or not^13^; that is, the sole presence of the condition or predisposition—which one might call the “disease”—does not indicate certainty that outcome will occur^13^ (Figure 1B).

Thus, in the situations when probabilities and outcomes are not independent, we can calculate the threshold that relates to the question: “For which values of parameters of both probabilities and utilities should we administer a treatment when we are certain in diagnosis/disease?” This will occur under generic definitions of net benefits and harms when *B* ≥ 0. Expressing it in specific terms, when morbidity due to disease (*M*) is used to define disutilities, the solution of the model shown in Figure 1B generates the following simple equation (see below and Appendix):

(2) $$M_{t}=\frac{H_{\mathrm{rx}}}{\mathrm{RRR}},$$

where *M*_*t*_ is the threshold for morbidity (outcome or event) in the absence of treatment and above which treatment should be given and below which should not be given; H_rx_—treatment‐related harms; RRR—efficacy (relative risk reduction) of treatment. Note that the model Figure 1B (Equation 2) assumes that the test that defines disease (the disease being a predisposition to subsequent morbid or mortal events) has already been performed (see Clinical Application below).

Therefore, we can use a decision model shown in Figure 1 to generate two threshold models: (1) when the probability of disease and probability of outcomes are independent of each other (and under assumption that probability of diagnosis is uncertain [0 ≤ *p* ≤ 1]; this yields calculation of *P*_*t*_ (Equation 1) and (2) when the probability of disease and probability of outcomes are not independent of each other (when we assume that diagnosis, ie, *P* = 1, this yields calculation of *M*_*t*_ (Equation 2).

### 2.4 Integration of a decision‐maker's preferences into the threshold model

The models outlined above assume that a decision‐maker weighs equally outcomes related to benefits and harms of treatments. Clinically, this is often not true. In the Clinical Application section, we will illustrate the calculation of the thresholds when a decision‐maker (eg, patient) weighs benefits and harms of treatments equally and differently.

To illustrate the role of patient's preference, we will express generic net benefits and harms through disutilities related to morbidity or mortality of treatment (*M*). We will also assume that most medical interventions express constant (relative) effects over the range of predicted absolute risk (often termed the “baseline risk”) and depending on the risk magnitude are conveniently modelled in decision analyses as odds ratio, risk ratio (RR), or *RRR* = 1 − *RR*. 14, 15 This allows intuitive interpretation of treatment effect: *p* · (1 − *RRR*); *RRR* = 1 means that the occurrence of outcome of interest is completely preventable (as *P* [ 1 – *RRR*] = 0), whereas *RRR* = 0 means that treatment does not affect underlying risk (*P* · [1 – *RRR*] = *P*).^14^, ^15^

We also introduce a variable *RV*_*H*_ to represent patient's (or decision makers') preferences expressed as relative values of harm of treatment with respect to the consequences of disease outcome *M* (when outcomes are equally valued, this is set at 1). If we now solve generic Equation 1 (applicable to the situations when probabilities and utilities are independent), using these specific definitions of benefits and harms (see Appendix for details), we obtain the following equation under EUT for the patient's threshold:

(3) $$P_{t}=\frac{{\mathrm{RV}}_{H}\cdot H_{\mathrm{rx}}}{\mathrm{RRR}\cdot M}.$$

This equation gives the threshold for the probability of diagnosis of disease at which the rational patient with preferences expressed as *RV*_*H*_ will be indifferent between accepting treatment versus not; that is, the patient will use treatment if the estimated probability of disease, *P* > *Pt*.

For decision when probabilities and outcomes are not independent of each other, as in Equation 2, we substitute *P* = 1 in the expressions above and solve for the parameter of interest. For example, we should administer treatment if risk of mortality or morbidity (*M*) without treatment is larger than the threshold:

(4) $$M_{t}=\frac{{\mathrm{RV}}_{H}\cdot H_{\mathrm{rx}}}{\mathrm{RRR}}.$$

Note that it is also impossible to know the value of *Hrx* and RRR in any individual patient as these events in each case will occur in the future. Hence, a decision in individual patients has to be based on the group (trial) data, ideally using multivariable risk prediction models.^16^

Note also that it is always the case that *M*_*t*_ ≤ *P*_*t*_, which is likely one of the reasons why actual decision‐making behavior has been observed to differ from the postulated normative behaviour (see Discussion).

## 3 APPLICATIONS

We illustrate the application of a revised threshold model to a common medical problem: recurrent VTE (rVTE). That is, a disease of interest is VTE, which is associated with substantial morbidity in terms of recurrent rethromboembolic outcomes or events (thus, percentage of the recurrent rethromboembolic outcomes are morbidities or disutilities used in our model). Note, however, that diagnosis of rVTE is made only after imaging shows the presence of new clot in deep veins or lungs.^17^ Another way to express this is that the “disease” is the predisposition to recurrent DVT that exists when a patient has experienced a prior DVT. Thus, in this case (and in the vast class of diagnostic problems), if one considers diagnosis is equal to VTE event (rather than the predisposition for having the event), the result would be double counting. As previously discussed, to avoid it, we set *P* = 1 and calculate threshold for rVTE outcome (see Equations 2 and 4).

We will analyse two clinical problems:

1. At which threshold for the probability of VTE disease do benefits of anticoagulation outweigh its bleeding risks to justify administration of anticoagulant treatments (Rx) over no treatment (No Rx)? This, as explained above, should be done only if the probability of disease and probability of outcomes are independent of each other.
2. At which threshold for the probability of recurrence for VTE does benefits of anticoagulation outweigh its bleeding risks to justify administration of anticoagulant treatments (Rx) over no treatment (No Rx)? This, as detailed earlier, is applicable to a type of clinical problems where probability disease and probability of outcomes are not independent of each other—indeed, when the patient is defined as already having the “disease” of interest and the issue is whether the patient will suffer the adverse events associated with the “disease.”

VTE, which consists of DVT and PE, is a common cause of morbidity and mortality in the United States; annually, about one in 120 people develops VTE.^18^ Once patients develop VTE disease, they are typically treated with anticoagulants, drugs that are effective in preventing complications of VTE such as PE and further VTE recurrence or reembolization (morbidity, event, or outcome of interest). However, anticoagulants are also associated with bleeding complications that may result in major morbidities or even death.

There is a widespread consensus based on randomized trials that have demonstrated a very high‐recurrence rate over 3 months in untreated or minimally treated patients and a very high RRR with treatment, that once patients develop VTE, barring obvious contraindications such as overt bleeding, rational patients will always choose treatment with anticoagulants for at least for 3 and perhaps 6 months.^19^ After 3 to 6 months, the probability of recurrence decreases substantially, and thus, the continuation of treatment beyond 3 months depends on the estimated risk of VTE recurrence versus bleeding risk.

As explained, from the modelling perspective, we can ask two questions: at which probability of VTE disease benefits of anticoagulation therapy outweigh its risks of bleeding. This is a different question from the one asking at which risk of VTE recurrence benefits of anticoagulation therapy outweigh its risks of bleeding. The former question can be answered only if the probability of disease and probability of outcomes are independent of each other (eg, probability of VTE and utility defined through mortality), while the latter should be used when outcome is a part of definition of disease, as in the case of rVTE disease, which is defined by very outcomes treatment is designed to prevent.^17^

Importantly, many practice guidelines and most authors consider VTE recurrence risk (at 5 years) exceeding 30% as high risk, intermediate as VTE recurrence of 15%, and low risk at less than or equal to 3% VTE.^19^, ^20^, ^21^, ^22^, ^23^ However, it is not clear (1) how these thresholds were calculated and (2) that considerations were given to the issues we discuss in this paper. In addition, although these authors recommend considerations of patients' V&P in determining when to prescribe anticoagulant treatments, they do not specifically show how that can be integrated into decision‐making at bedside.^19^, ^20^, ^21^, ^22^ Other investigators have developed decision aids that are extremely specific in demonstrating how values and preference can be integrated in decision‐making. One of our goals in this paper is to compare the calculated thresholds with these (descriptive) thresholds commonly used in practice with and without taking V&P into account.^24^

### 3.1 Data

The EINSTEIN investigators tested efficacy of rivaroxaban (drug that belongs to class of direct oral anticoagulants [DOAC]) versus placebo for secondary prevention of VTE.^25^ The decision regarding treatment was made after patients who were eligible for the trial had objectively confirmed, symptomatic DVT or PE using ultrasound, or lung imaging^25^; that is, the patients with confirmed symptomatic DVT or PE who had been treated for 6 or 12 months with a vitamin K antagonist or rivaroxaban were then randomly assigned to receive continued treatment with rivaroxaban or placebo.^25^ The primary efficacy outcome was symptomatic, rVTE, defined as the composite of DVT or nonfatal or fatal PE.^25^ We model the situation when no further testing is possible and do not model clinical suspicion whether test should be done—the latter is a purview of the classic threshold model.^1^, ^9^

The EINSTEIN investigators found that over 6 to 12 months treatment duration, in the placebo group, the proportion of patients experiencing VTE recurrence was $\;M=\frac{42}{594}=7.1\%.$ In the group being treated with rivaroxaban, the proportion of patients with VTE recurrence was $\;M_{\mathrm{rx}}=\frac{8}{602}=1.3\%.$ Therefore, $\mathrm{RRR}=\frac{M-M_{\mathrm{rx}}}{M}=81.2\%$ (this translates into absolute risk reduction of $\;\frac{42}{594}-\frac{8}{602}\approx 0.071-0.013=5.8\%$). However, rivaroxaban is also associated with an increase risk of major bleeding *H*_*rx*_ = 4.8 %.

1. Calculation of the probability of VTE disease/diagnosis at which we should administer rivaroxaban:
  We will first assume that a decision‐maker (eg, patient) may value consequences of bleeding equally to consequences of VTE. To simplify calculations, we express these preferences as relative values toward bleeding outcomes with respect to VTE outcomes, which we set at 1.
  Assuming that the probability of diagnosis of VTE and recurrence of VTE are independent of each other, we could calculate that the threshold probability for diagnosis of VTE ( $T_{dx_{\mathrm{VTE}}}$) at which we are indifferent between giving rivaroxaban versus placebo is given by the Equation 3 above as follows:
  $$T_{dx_{\mathrm{VTE}}}=\frac{{\mathrm{RV}}_{H}\cdot H_{\mathrm{rx}}}{\mathrm{RRR}\cdot M}=\frac{1\cdot 0.048}{0.812\cdot 0.071}=83\%.$$

These, however, implausibly high thresholds are not observed in practice and are not recommended by guidelines panels. For the reasons discussed in this paper, this answer is also not normatively correct^12^ because “disease” was defined as the outcome of VTE rather than as the predisposition to rVTE created by the first VTE.^25^ Normatively accurate answer could be obtained if we considered the outcome of mortality rather than rVTE. For example, the EINSTEIN investigators reported death due to VTE and bleeding of 1/602 (0.2%) and 0/602 (0%), respectively.^25^ Using these values to calculate the threshold probability for diagnosis of VTE ( $T_{dx_{\mathrm{VTE}}}$) above which we should give rivaroxaban (vs placebo), we obtain the following:

$$T_{dx_{\mathrm{VTE}}}=\frac{{\mathrm{RV}}_{H}\cdot H_{\mathrm{rx}}}{\mathrm{RRR}\cdot M}=\frac{1\cdot 0}{0.812\cdot 0.002}=0\%,$$

which is normatively but not descriptively correct (as no patients would choose to use rivaroxaban to prevent death when the probability of dying without rivaroxaban over the relevant time period was zero).^19^, ^20^, ^21^, ^22^

- Calculation of the probability of recurrence of VTE (*M*) at which we should administer rivaroxaban:
  If there is dependence between probability and outcomes indicating that we are certain in diagnosis/disease (ie, we define diagnosis/disease as the predisposition to recurrence created by the first event), the threshold for the value of morbidity or mortality is given by formula (4) above.
  $$M_{\mathrm{VTE}}=\frac{{\mathrm{RV}}_{H}\cdot H_{\mathrm{rx}}}{\mathrm{RRR}}=\frac{1\cdot 0.048}{0.812}=5.9\%$$

**Figure 2:**
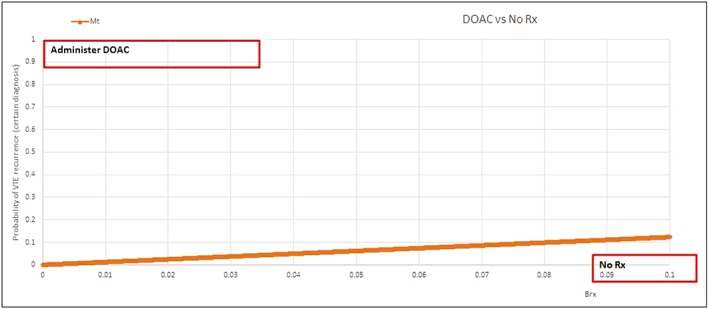
When to give novel anticoagulant (direct oral anticoagulants) for prevention of venous thromboembolism versus no treatment? The direct oral anticoagulants should be given if the probability of recurrent venous thromboembolism event (outcome) is above the calculated threshold line for given bleeding risk (Brx) (shown on *x*‐axis) (NB the calculation assumes that a decision‐maker considers avoiding venous thromboembolism and bleeding equally important)

In other words, if we use the disease definition above, we should administer rivaroxaban only if the probability of recurrence of VTE (without treatment) is above 5.9%.Note that in this case, utility threshold falls within a range that could be descriptively correct. Thus, differences between threshold based on comparison of the VTE recurrence with the threshold based on the probability of VTE disease can explain the differences between what is observed in practice and what original threshold model prescribed. Figure 2 illustrates *M*_*t*_ as a function of bleeding risk.

### 3.2 Integration of patients' values and preferences

Empirical data indicated that most patients value bleeding event about three fourth as bad as VTE event,^26^ which is to say that most patients value avoiding a clot 1.3 (=1/0.75) times more than avoiding bleeding. However, the range of values dramatically vary from tolerating no excess of bleed to prevent a clot to accepting 22 times excess of bleeds to avoid a clot (*RV*_*H*_ = 0.045) to valuing them equally.^27^ For our baseline analysis, if we set *RV*_*H*_ = 0.75, the threshold for the probability of VTE recurrence is MVTE=RVH·HrxRRR=.0.75·0.0480.812=4.4%.

**Figure 3:**
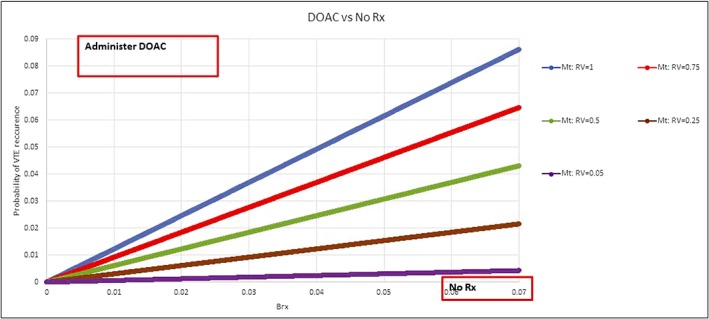
Sensitivity analysis displaying calculations of the decision thresholds as a function of given bleeding risk (Brx) for five values of patient's preferences with respect to consequences of bleeding versus venous thromboembolism: *RV*_*B*_: 1 (bleeding and venous thromboembolism are equally weighted), 0.75, 0.5, 0.25, 0.05 (avoiding venous thromboembolism is 1.3, 2, 4, and 20 more times important than avoiding major bleeding, respectively. The direct oral anticoagulants treatment should be given if the probability of recurrent venous thromboembolism event (outcome) is above the calculated threshold line for given Brx (shown on *x*‐axis)

If we vary values for bleeding outcome from *RV*_*H*_ = 0.045 (VTE is 22 times worse than bleeding) to *RV*_*H*_ = 1 (VTE is equally weighted as bleeding), the threshold for the probability of VTE recurrence ranges from *M*_*VTE*_ = 0.3% to *M*_*VTE*_ = 5.9 % , which does encompass the ranges recommended by practice guidelines (Figure 3).^19^, ^20^, ^21^, ^22^

## 4 DISCUSSION

The threshold model is probably one of the most important advances in the field of medical decision‐making,^1^, ^9^ which links evidence (which exists on the continuum of credibility) with decision‐making (which is a categorical exercise—we decide to act or not to act).^2^ The model is not, however, widely used by clinicians at bedside even though it addresses extremely common classes of the medical problems and despite the fact that nomograms to facilitate its use were published more than two decades ago.^28^ The model has also not attracted deserved attention even if it is likely that physicians act according to their different thresholds, which, at least in part, can probably explain the tremendous variation observed in today's practice of medicine.^2^, ^29^

One of the common explanations provided for the lack of popularity of the threshold models among practitioners is that the original models were based on EUT, which is known to be widely violated both by lay people and physicians^2^, ^3^, ^5^; that is, physicians do not act at the EUT prescribed thresholds, but rather below or above it,^5^, ^30^, ^31^, ^32^ depending on the context and theoretical approach, which can better explain the observed behaviour.^3^, ^4^ This has prompted reformulation of the threshold model using different theoretical frameworks such as regret and dual‐processing theory model.^2^, ^3^, ^5^, ^30^, ^33^, ^34^ Indeed, some empirical evidence shows that thresholds at which physicians act are more consistent with regret and dual‐processing theory model than with the EUT model.^5^, ^35^

However, in this paper, we provide a considerably more compelling explanation of why normative recommendations based on the EUT threshold model may differ from the actual decisions seen in practice. First, considerable confusion regarding definitions of “disease” have existed: In considering issues of prevention, patients and physicians are considering the “disease”—or perhaps more appropriately the condition—as a biological predisposition to subsequent morbid or mortal events. Another way to state this is that calculation of the threshold probability of diagnosis is normatively correct only when health outcomes are not part of definition of disease. Otherwise, the calculations are not correct because of value‐induced bias^12^—violation of the requirement that probabilities and utilities are independent of each other.

When disease is defined as present at the time of decision‐making, ie, clinicians are considering patients as having a biological predisposition to subsequent adverse events—in the original threshold model, they have a 100% probability of being “diseased” or, to put it another way, when health outcomes that treatment are designed to affect are part of disease definition, we showed that the proper way to use threshold model is to calculate the threshold for utilities, ie, the threshold for the probability of outcome or risk of event (and not diagnosis) at which we should administer treatment.

Differentiating between situations in which the issue is whether disease is present and adverse outcomes will only occur if it is, from one in which all patients are at risk of adverse outcomes, has also important implications for recommendations about use of diagnostic tests. The second classic threshold model^9^ recommends calculating two thresholds: the testing and the test‐treatment thresholds by taking test sensitivity and specificity into consideration. The role of a test is to increase diagnostic certainty, but when diagnosis—the predisposition to adverse events—is certain, calculation of probability of diagnosis is nonsensical. Thus, when probability of disease and probability of outcomes are not independent of each other, diagnostic test characteristics such as sensitivity and specificity should not be taken into consideration, but instead, recommendations should be based on calculations of *M*_*t*_ as illustrated in this paper.

Our second explanation of differences between normative and descriptive findings is that the original threshold model did not take a decision‐maker's V&P explicitly into account.^6^ However, when the effect of V&P is incorporated in the threshold model, it actually can be descriptively correct (Figure 3). The previous criticism of the classic threshold model revolved about descriptively unrealistic low thresholds prompting reformulation of the threshold equations from non‐EUT theoretical frameworks.^2^, ^31^, ^34^, ^35^ However, we found that *M*_*t*_ ≤ *p*_*t*_, ie, even lower than the thresholds based on the original threshold model. Yet, in the context of our VTE example, taking V&P into account align the threshold model quite well with the current practice guidelines, which recommend administration of treatment when the VTE recurrence risk (at 5 years) exceeds 30%, 15%, and 3% for high, intermediate, and low risk of VTE recurrence, respectively.^19^, ^20^, ^21^, ^22^ This makes descriptive sense because when physicians and patients perceive that diagnosis is certain, they are more inclined to act than when diagnosis is not certain. As clear as these recommendations about the threshold are, typically guidelines panels have not actually explicitly taken patients' V&P into calculations of the thresholds—although there is at least one notable exception, the ninth iteration of the American College of Chest Physicians Antithrombotic Guidelines (which conducted a systematic review of V&P for antithrombotic therapy^36^ and specified an equal importance to serious bleeding rVTE).^37^ Nevertheless, despite typically neglecting to show how thresholds are calculated, guidelines panels do routinely make such recommendations.^19^, ^20^, ^21^, ^22^

Thus, under some considerations, EUT appears to be descriptively realistic. In this context, Felder and Mayrhofer^38^ argued the descriptive power of EUT can be further augmented if treatment effects are modelled directly on the probability of disease^14^, ^15^ not in utilities as in the original threshold model.^1^, ^9^ In a separate (forthcoming) paper, we showed that regardless how the effect of treatment is modelled, the model yields identical results under EUT but not under regret theory.^30^, ^31^, ^32^ However, we also consider the question which decision theory (EUT vs non‐EUT) is more descriptively accurate an empirical question.^5^ We have recently proposed that “one size does not fit all,” ie, there cannot be one theory of rationality that can meet all our needs in all contexts.^4^ Hence, we should abandon debate if the EUT is superior to the non‐EUT or vice versa; rather, we should define the circumstances when application of one theory is more suitable to use than the other.

Finally, many consider inadequate decision‐making as one of the major culprits for today's suboptimal patient outcomes^39^, ^40^, ^41^, ^42^ to the point that some decision scientists have suggested that personal decisions are the leading cause of death.^43^ Promoting the threshold model, which was introduced more than 40 years ago^1^ and which offered a simple but powerful yet neglected tool, may considerably improve inadequate decision‐making often seen in todays' practice.^43^ As illustrated in this paper, however, clinicians and guideline developers have to ensure they appropriately apply the threshold model.

**Figure 4:**
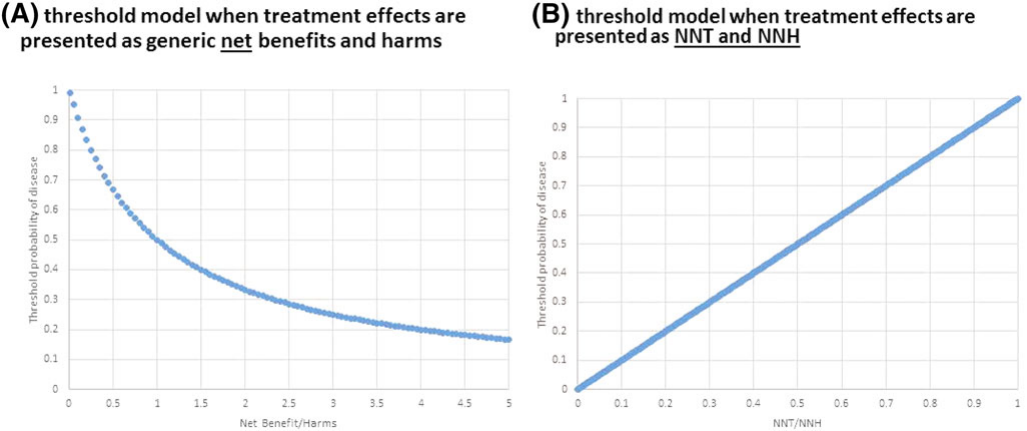
An illustration of the differences in calculation of the threshold probability as a function of definition of treatment effects: A, Threshold as a function of generic net benefits and harms as originally proposed by Pauker and Kassirer,^1^ see equation (1) B, threshold as a function of evidence‐based treatment metrics (Pt=NNT/NNH) originally proposed in other study^6^ (the model assumes that probabilities and utilities are independent of each other; see Figure 1A and text for details)

The problems we discussed in this paper have arisen from misunderstanding one of the key aspects of P&K^1^ model to which we draw attention in this article: The threshold calculation depends on the definition of disease.^6^ We demonstrate here that the key to proper application of the threshold model is to understand if the condition (or disease) of interest is the predisposition to subsequent adverse events—or to put it another way, if outcomes that are part of the definitions of treatment benefits and harms are also integral aspect of the definition of disease that we wish to model—or if it is not. In addition, use of generic definitions of treatment benefits and harms as per original model^1^ as opposed to widely accepted EBM clinical measures of treatment effects^6^ has created misunderstanding about true values of the threshold at which treatment should be administered. Figure 4, which shows how threshold probability of diagnosis dramatically differs when it is expressed as generic net benefits and harms versus popular evidence‐based measures of treatment effects (number of patients needs to be treated in order for one patient to benefit)/ (number of patients who need to be exposed to treatment in order for one patient to be harmed) (NNT/NNH), illustrates this distinction.^6^ Obviously, calculation of the threshold depends on the accuracy of the parameters that are used to populate the model. Ideally, the data for calculation of the threshold should be based on a well‐done systematic reviews/meta‐analyses; this is where decision analysis meets evidence‐based medicine.^6^ Here, it is important to note that even though our model is meant to help individualize treatment decisions, ultimately data to populate the model are based on average, group estimates. Indeed, risk is a group phenomenon and is knowable and accurately measured as a population‐based measure.^13^, ^44^ We can never say with perfect certainty which individual patient will develop the outcome of interest^13^, ^44^; that is, risk in any individual patient remains ultimately unknowable.^44^ However, we simply do not have better way to individualize our treatments but to rely on the risk information from the groups.^13^, ^44^, ^45^ In fact, one can argue that the entire goal of personalized and precision medicine is to reliably reduce the population to smaller groups, in which risk can still be assessed with high accuracy that may be better applicable to individuals.^44^

From the perspective of this paper, more systematic assessment of application of the threshold models in clinical practice would be desirable. In particular, alignment of recommendations by the guidelines panels with the threshold model calculations would improve clinical practice guidelines. Wider application of the one of most significant development in history of decision‐making may go long way to help practitioners and patients improve their decisions.

## APPENDIX A

### Modelling treatment effect: Treatment versus no treatment

In the classic P&K model^1^ (Figure 1), the expected utilities of each decision are given as follows:

$$\mathrm{EU}\left[\mathrm{Rx}\right]=p\cdot U_{1}+\left(1-p\right)U_{2}\;\mathrm{EU}\left[\text{NoRx}\right]=p\cdot U_{3}+\left(1-p\right)U_{4}.$$

Following Pauker and Kassirer,^1^ we define the net benefit of treatment as *B* = *U*_1_ − *U*_3_, ie, the difference in the utility of the outcomes if the diseased patient was treated or was not treated and net harms as *H* = *U*_4_ − *U*_2_, ie, the difference in the utility of the outcomes if a patient without the disease was not treated versus treated. Solving the tree, the threshold probability (*P*_*t*_) above which we should treat is calculated as follows:

(1) $$P_{t}=\frac{1}{1+\frac{B}{H}.}$$

Equation 1 shows a generic version of the threshold equation based on generic definitions of net benefits and harms as per above. To obtain, specific version of the threshold equation, we substitute net benefits and harms through disutilities related to morbidity or mortality (*M*) associated with treating versus not treating (or using one treatment vs another). We distinguish *M*—morbidity or mortality that occur in the absence of treatment—and Mrx (morbidity/mortality that occur while on the treatment); we also define Hrx as harms that occur due to treatment.

We also assumed that most medical interventions have constant (relative) effects over the range of predicted absolute risk and are conveniently modelled in decision analyses as *RR* or *RRR* = 1 − *RR*. 14, 15 This allows intuitive interpretation of treatment effect: *P* · (1 − *RRR*); *RRR* = 1 means that the occurrence of outcome of interest is completely preventable (as *P* [ 1 – *RRR*] = 0), whereas *RRR* = 0 means that treatment does not affect underlying risk (*P* · [1 – *RRR*] = *P*).^14^, ^15^ We also introduce a variable *RV*_*H*_ to represent patient's (or decision makers') preferences expressed as relative value of avoiding harms of treatment, *H*_*rx*_ with respect to avoiding disease outcome, *M*.

Thus, we express (dis)utilities in the following way:

*U*_1_ = *U*(*Rx*, *D*+) = 1 − *M*_*rx*_ − *RV*_*H*_ · *H*_*rx*_ = 1 − *M* · (1 − *RRR*) − *RV*_*H*_ · *H*_*rx*_ ^*^ [Patients with disease on Rx]
*U*_2_ = *U*(*Rx*, *D*−) = 1 − *RV*_*H*_ · *H*_*rx*_   [Patients without disease on Rx: **overtreatment**]
*U*_3_ = *U*(*NoRx*, *D*+) = 1 − *M*   [Patients with disease not on Rx: **undertreatment**]
*U*_4_ = *U*(*NoRx*, *D*−) = 1  [**Healthy** patients, no disease, no treatment]

(Disutility represents undesirability, or strength of preferences that individuals or societies have against a particular outcome such as loss of length of life, mortality or morbidity rates [the latter often calculated by counting event or outcome of interest such as VTE recurrence etc], presence of pain, cost, etc, a complement of utility, typically expressed as 1—utility (desirability or strength of preferences that individuals or societies have for a particular outcome such as length of life, morbidity or mortality rates, absence of pain, cost, etc.])

Substituting these values into the definition of net benefit *B* = *U*_1_ − *U*_3_ = *M* · *RRR* − *RV*_*H*_ · *H*_*rx*_ and net harms *H* = *U*_4_ − *U*_2_ = *RV*_*H*_ · *H*_*rx*_, and assuming that the patient values avoiding treatment harms equally to avoiding effect of disease (ie, *RV*_*H*_=1), we obtain the following:

$$P_{t}=\frac{H_{\mathrm{rx}}}{\mathrm{RRR}\cdot M}.$$

This equation is valid if utilities of outcomes (expressed via *M*) are not used for definition of disease, ie, it assumes that the probability of disease and utilities are independent of each other (Figure 1a). * Mathematically a more correct representation of this utility has the following form:

$$U_{1}=U\left[\mathrm{Rx},D+\right]=1-M_{\mathrm{rx}}-RV_{H}\cdot H_{\mathrm{rx}}+\left[\;M_{\mathrm{rx}}\cdot H_{\mathrm{rx}}\right],$$

but we assumed that simultaneous occurring of effect of disease and harms of treatment is clinically rare occurrence and is also mathematically negligible, which is the reason we did not include this product in the definition.

If this condition stated in the equation above is not satisfied, as explained in the text, we modify the model by assuming *P* = 1 (Figure 1B). In that case, the expected values of Rx and NoRx are equal if the morbidity due to disease event is

$$M_{t}=\frac{H_{\mathrm{rx}}}{\mathrm{RRR}}.$$

If we assume a decision‐maker's values of avoiding harms of treatment, *H*_*rx*_ with respect to avoiding disease outcome, *M* is different (*RV*_*H*_), then the two formulas above are

$$P_{t}=\frac{{\mathrm{RV}}_{H}\cdot H_{\mathrm{rx}}}{\mathrm{RRR}\cdot M}\;\text{and}\;M_{t}=\frac{{\mathrm{RV}}_{H}\cdot H_{\mathrm{rx}}}{\mathrm{RRR}}.$$

### Treatment1 versus treatment2

If we want to calculate the threshold at which we want to select one treatment over another, we define utilities somewhat differently^6^:

$$\begin{array}{l} U_{1}=U\left(\mathrm{Rx}1,D+\right)=1-M_{\mathrm{rx}1}-{\mathrm{RV}}_{H}\cdot H_{\mathrm{rx}1}=1-M\cdot \left(1-\mathrm{RRR}1\right)-{\mathrm{RV}}_{H}\cdot H_{\mathrm{rx}1}\\ U_{2}=U\left(\mathrm{Rx}1,D-\right)=1-{\mathrm{RV}}_{H}\cdot H_{\mathrm{rx}1}\\ U_{3}=U\left(\mathrm{Rx}2,D+\right)=1-M_{\mathrm{rx}2}-{\mathrm{RV}}_{H}\cdot H_{\mathrm{rx}2}=1-M\cdot \left(1-\mathrm{RRR}2\right)-{\mathrm{RV}}_{H}\cdot H_{\mathrm{rx}2}\\ U_{4}=U\left(\mathrm{Rx}2,D-\right)=1-{\mathrm{RV}}_{H}\cdot H_{\mathrm{rx}2} \end{array}.$$

Note that we assume that the patient will value harms of treatment relative to morbidity of disease (*RV*_*H*_) in the same way regardless if it was caused by treatment 1 versus treatment 2 (it is, of course, possible to assume different *RV*_*H*_, but we consider this outside of a scope of this paper).

Solving these equations for the threshold *P*_*t*_ (ie, assuming that the probability of disease and utilities are independent), we get

$$P_{t}=\frac{{\mathrm{RV}}_{H}*\left(H_{\mathrm{rx}1}-H_{\mathrm{rx}2}\right)}{M_{\mathrm{rx}2}-M_{\mathrm{rx}1}}=\frac{{\mathrm{RV}}_{H}*\left(H_{\mathrm{rx}1}-H_{\mathrm{rx}2}\right)}{M*\left(\mathrm{RRR}1-\mathrm{RRR}2\right)}.$$

If the assumption of independence between utilities and probabilities is violated, similar to above, we obtain

$$M_{t}=\frac{{\mathrm{RV}}_{H}\cdot \left(H_{\mathrm{rx}1}-H_{\mathrm{rx}2}\right)}{\left(\mathrm{RRR}1-\mathrm{RRR}2\right)}.$$

If we want to model effects of the multiple harms, we can write

$$\begin{array}{l} H_{\mathrm{rx}1}={\mathrm{RV}}_{H}*H_{\mathrm{rx}11}+{\mathrm{RV}}_{H}*H_{\mathrm{rx}12}+\ldots +{\mathrm{RV}}_{H}*H_{\mathrm{rx}1m}\\ H_{\mathrm{rx}2}={\mathrm{RV}}_{H}*H_{\mathrm{rx}21}+{\mathrm{RV}}_{H}*H_{\mathrm{rx}22}+\ldots +{\mathrm{RV}}_{H}*H_{\mathrm{rx}2n} \end{array}.$$

Then, simply replacing *H*_*rx*1_ and *H*_*rx*2_ in the equations above, we can calculate the thresholds based on considerations of multiple harms.
